# Science and the Soul

**Published:** 1911-10-28

**Authors:** 


### SCIENCE AND THE SOUL.

PROFESSOR HENRI BERGSON's philosophy has lately attracted considerable attention, and his second lecture last week at Cambridge, wherein he dwelt at some length on his conception of the Nature of the Soul, has been extensively reported. At first glance it may seem that so abstruse and hypothetical a subject as that which the Belgian professor took as his theme has but little medical interest, whatever academic interest it may hold for the general scientist. But the new—or rather, old redressed—conception that is put forward in Professor Bergson's books, now so well known, and which was further illuminated in last week's popular lecture, has undoubtedly some aspects on which the medical man is as competent to judge as the average philosopher. It holds other aspects, also, which the former is much more able to criticise knowledgeably than the general run of philosophers. The intangibility of the subject, the disheartening *non possumus* that has awaited every investigator from Shentung to Fechney, and above all the want of experimental data of a nature sufficiently reliable to be convincing even in a minor degree, have hitherto discouraged the medical philosopher, even when so expert and well qualified as were Paracelsus and Boerhaave, from devoting himself wholeheartedly to the consideration of this fascinating subject. But, as the philosopher doctor of Salzburg remarks in his rhapsodical tirade against the brutal arrogance of contemporary science, intangibility and mystery are not necessarily presumptions in favour of non-existence, and the "matters with which the medical man has to deal are so mysterious that he may well think others, a tithe less so, equally worthy of his mind.

Yet the invitation has not been taken up. Science has been curiously silent about the soul. It has treated the world to far-fetched theories of immunity which doubtless a century hence will seem as ludicrous to scientists of the day as Cagliostro's pretensions to immortality appear to modern men of common-sense. It has evolved theories of etiology, pathology, and nosology which for subtlety and innate predilection for hair splitting reminds the philosopher of the disquisitions of the quodlibetic schoolmen of the age of Aquinas. But it has never seriously set itself to consider, scientifically, the subject which philosophers, without the knowledge that medical men possess, have attacked not altogether fruitlessly. Probably the reason is that the subject is still regarded as untouchable in the laboratory or the hospital ward. Questions of abstract philosophy, especially those which touch matters round which the sentiment, religious and moral, of ages have shed a halo of sanctity, do not lend themselves to the calm discussion which must be the preface to any scientific conclusion. At the same time, they do not lend themselves readily to destructive philosophical treatment. Yet the latter has been attempted, and philosophy is all the better for it. In the circumstances it is worth while for a serious scientist to give us his conception of the soul—a conception not made up of the broken fragments of former fancies such as M. Bergson submits, nor one of stubborn negation of whatever is incapable of direct and material proof as Dekker gave, nor yet the somewhat weird conception framed by Mr. Myers and, thanks to the efforts of theosophists and psychicists, now so popular among a certain class, but one which realises that the problem of the soul must be attacked from a scientific as well as from a purely philosophical point.

